# Fra-2 overexpression upregulates pro-metastatic cell-adhesion molecules, promotes pulmonary metastasis, and reduces survival in a spontaneous xenograft model of human breast cancer

**DOI:** 10.1007/s00432-021-03812-2

**Published:** 2021-10-24

**Authors:** Sabrina Arnold, Jan Kortland, Diana V. Maltseva, Stepan A. Nersisyan, Timur R. Samatov, Susanne Lezius, Alexander G. Tonevitsky, Karin Milde-Langosch, Daniel Wicklein, Udo Schumacher, Christine Stürken

**Affiliations:** 1 Institute of Anatomy and Experimental Morphology, University Cancer Center Hamburg, University Medical Center Hamburg-Eppendorf, Martinistrasse 52, 20246 Hamburg, Germany; 2 Faculty of Biology and Biotechnology, National Research University Higher School of Economics, Myasnitskaya Str. 13/4, 117997 Moscow, Russia; 3 Scientific Research Center Bioclinicum, Ugreshskaya Str. 2/85, 115088 Moscow, Russia; 4 Evotec International GmbH, Marie-Curie-Str. 7, 37079 Göttingen, Germany; 5 Department of Medical Biometry and Epidemiology, University Medical Center Hamburg-Eppendorf, Martinistrasse 52, 20246 Hamburg, Germany; 6 Department of Gynecology, University Medical Center Hamburg-Eppendorf, Martinistrasse 52, 20246 Hamburg, Germany

**Keywords:** Breast cancer, Transcription factor, AP-1, Fra-2, Metastasis

## Abstract

**Purpose:**

The transcription factor Fra-2 affects the invasive potential of breast cancer cells by dysregulating adhesion molecules in vitro. Previous results suggested that it upregulates the expression of E- and P-selectin ligands. Such selectin ligands are important members of the leukocyte adhesion cascade, which govern the adhesion and transmigration of cancer cells into the stroma of the host organ of metastasis. As so far, no in vivo data are available, this study was designed to elucidate the role of Fra-2 expression in a spontaneous breast cancer metastasis xenograft model.

**Methods:**

The effect of Fra-2 overexpression in two stable Fra-2 overexpressing clones of the human breast cancer cell line MDA MB231 on survival and metastatic load was studied after subcutaneous injection into scid and E- and P-selectin-deficient scid mice.

**Results:**

Fra-2 overexpression leads to a significantly shorter overall survival and a higher amount of spontaneous lung metastases not only in scid mice, but also in E- and P-deficient mice, indicating that it regulates not only selectin ligands, but also selectin-independent adhesion processes.

**Conclusion:**

Thus, Fra-2 expression influences the metastatic potential of breast cancer cells by changing the expression of adhesion molecules, resulting in increased adherence to endothelial cells in a breast cancer xenograft model.

**Supplementary Information:**

The online version contains supplementary material available at 10.1007/s00432-021-03812-2.

## Introduction

Breast cancer is the most frequent form of cancer in women and the most common cause of female cancer death (Eccles et al. 2013). Metastasized breast cancer still represents a considerable clinical problem, as it cannot ultimately be cured and thus most patients die because of the metastatic spread to vital organs such as lung, bone marrow, and liver (Pantel and Brakenhoff 2004). To achieve progress in the treatment of metastasized breast cancer, a better understanding of the process of metastasis formation is therefore necessary. During distant metastasis formation, cancer cells first of all separate from the primary tumour and invade the local stroma, enter the tumour blood vessels, and are transported through the circulation to distant sites. Here, adhesion to endothelial cells and extravasation into the stroma of the secondary site follows, where some of the emigrated cells eventually grow into clinically detectable metastases (Carr and Orr 1983; Fidler 1978, 2003). Endothelial cells at this site express E- and P-selectins at their luminal surface, which have been previously described as mediators of the first step of leucocyte adhesion and transmigration at sites of inflammation. Comparable with leucocyte adhesion, studies have demonstrated an involvement of selectins in the attachment and transmigration of circulating tumour cells (Gout et al. 2008; Tremblay et al. 2006). The particular contribution of selectin expression to cancer progression is defined by the temporal and spatial presence of selectins on the luminal endothelial cell surface but also on the presence of selectin ligands on the cancer cell membrane (Borsig 2018). The classical (canonical) binding partners of selectins are carbohydrate structures, which must be presented on specialized protein scaffolds that enhance ligand clustering, which enables effective selectin binding (Varki 1997). Enhanced selectin ligand expression on tumour cells enables the contact with the endothelium and thereby regulates tumour cell progression (Borsig 2018).

In a foregoing study, we demonstrated that the overexpression of the transcription factor Fra-2 (FOSL2 or Fos-related antigen-2) in MDA MB231 cells resulted in altered in vitro expression of cell-adhesion molecules involved in cell–cell or cell–matrix interactions and investigated the adhesive potential of these transfected cells. A strong increase of transiently adherent (rolling) cells compared with the control cells was observed in laminar flow assays with E-selectin coated surfaces, indicating that Fra-2 overexpression upregulates expression of cell-adhesion molecules or their ligands, respectively, of the leukocyte adhesion cascade (Schroder et al. 2010). In particular, the observation that increased Fra-2 expression can lead to increased attachment of the cancer cells to E-selectin suggests that Fra-2 also modulates selectin-binding sites in breast cancer cells in vivo and thus allows the tumour cells to adhere to the vascular endothelium via E-selectin.

The expression of these cell-adhesion molecules and their ligands underlies the regulation by transcription factors including Fra-2. Fra-2 (Fos-related antigen-2) is part of the AP-1 (activating protein-1) transcription factor complex, consisting of the Fos-, Jun-, ATF-, and MAF-protein families (Angel and Karin 1991; Eferl and Wagner 2003; Goetz et al. 1996; Vogt and Bos 1990) that bind to regulatory sequences of various target genes, which in turn are induced by growth factors, cytokines, oncoproteins, or tumour promoters like TPA (12-*O*-tetradecanoylphorbol-13-acetate). They form hetero- or homodimers, whose respective combination determines the genes that are regulated by AP-1 (Eferl and Wagner 2003). Initially, Fra-2 was thought to be primarily involved in bone formation (Bozec et al. 2013) and appeared to be associated with several different physiological and pathological processes, such as photoperiodic regulation (Engel et al. 2005) or fibrosis (Roy et al. 2010). In addition, further studies have shown that Fra-2 might play a pivotal role in the progression of diverse human tumour types in vivo (Milde-Langosch 2005). Fra-2 overexpression was found in salivary gland tumours (Maruya et al. 2004), colorectal cancer (Zhang et al. 2005), adult T-cell leukaemia (Nakayama et al. 2008), cutaneous T-cell lymphomas (Nakayama et al. 2012), and tongue cancer (Gupta et al. 2015). Although breast cancer is probably the most intensively studied tumour entity in terms of the role of AP-1 members in malignant progression, still considerably uncertainties about the function of Fra-2 expression in breast cancer exist. So far, only one study has shown that in breast cancer, overexpression of Fra-2 is associated with a more aggressive tumour phenotype, suggesting that Fra-2 may be involved in breast cancer progression in vivo (Milde-Langosch et al. 2008).

It was also recently shown that Fra-2 facilitates TGF-ß1-induced migration in non-small cell lung cancer (NSCLC) cells by interaction with the transcription factor Smad3 (Wang et al. 2014). Due to these multiple interactions, Fra-2 is an ideal candidate, which can possibly exert a regulatory effect on the expression of many different cell-adhesion molecules and their ligands, respectively. We therefore wanted to investigate which of these regulatory effects of Fra-2 operate in an in vivo model of breast cancer metastasis and focused on E- and P-selectin-binding sites as the first receptors of the leukocyte adhesion cascade. By studying the effect of Fra-2 overexpression in MDA MB231 breast cancer cells in scid mice and E- and P-selectin-deficient mice, we investigated the role of this transcription factor in tumor cell adhesion and metastasis in vivo. By comparison of tumor xenografts in both mouse models, we further analysed the role of selectins from both the ligand and the receptor sides during this process.

## Materials and methods

### Cell lines

All cells derived from the human breast cancer cell line MDA MB231 were cultivated as described before (Milde-Langosch et al. 2008) and stable clones with increased Fra-2 expression had been generated by transfections with the plasmid Fra-2-pIRES containing the full Fra-2 cDNA cloned in the bicistronic vector pIRES (Milde-Langosch et al. 2008). For the following experiments, the MDA-Fra-2 clones (cl) 1 and 2 and the MDA-pIRES clone as negative control were used. All cell lines were routinely tested for mycoplasma infection using the PCR-based VenorGeM Mycoplasma Detection kit (Minerva Biolaps GmBH, Berlin, Germany) according to the manufacturer’s instructions. Only cells negative for mycoplasma infection were used for further experiments. Authenticity of human cell lines has been confirmed by STR analysis (DSMZ Braunschweig, Germany).

### Subcutaneous xenograft mouse model

In this study, female Balb/c severe combined immunodeficient (scid) mice as well as E-selectin −/− and P-selectin −/− scid mice were used. Scid mice had been crossbred with E- and P-selectin-deficient mice (Jackson laboratory, stock no: 002916) and selectin deficiency was verified as described previously (Stubke et al. 2012). The mice were housed under pathogen-free conditions in IVC cages and provided with sterile food and water ad libitum. For injection into mice cells from MDA-Fra-2 clone 1 and 2 and as negative control MDA-pIRES cells were adjusted to 5 × 10^6^ ml^−1^ medium. 200 µl of this suspension were injected subcutaneously between the scapulae of each mouse. Cells from every cell line were injected into groups of 20 animals each. When the primary tumours had reached maximum 20% of the body weight of the animal at the beginning of the experiment or ulcerated, the mice were terminally narcotized, and then sacrificed by cardiocentesis and cervical dislocation. Right lungs were excised en bloc and prepared for histologic analysis; left lungs were subjected to DNA isolation. Bone marrow was received by flushing the left femora with 1 ml NaCl 0.9%. Two hundred microliters of blood and the bone marrow suspension were subjected to DNA isolation. Then primary tumours were removed, weighed, and processed for histologic analysis, RNA isolation, and protein isolation.

### Ethical approval and informed consent

The methodology for carrying out the animal experiments was consistent with the UKCCCR guidelines for the welfare and use of animals in cancer research (Workman et al. 2010) and were carried out in compliance with the appropriate Animal Research: Reporting in vivo Experiment (ARRIVE) guidelines (Percie du Sert et al. 2020). The xenograft experiment was supervised by the animal welfare officer institute and the local licensing authority (Behörde für Soziales, Familie, Gesundheit und Verbraucherschutz, Amt für Lebensmittelsicherheit und Veterinärwesen, Hamburg, Germany) approved the experiment under the Project No. G09/58. All methods were performed in accordance with the relevant guidelines and regulations by the local authorities.

### Histology and morphological analysis of spontaneous lung metastases

One piece of primary tumour, right lung, and bone marrow were fixed in 4% buffered formalin and processed for wax histology. Five µm sections were cut from primary tumours for immunohistochemistry and hematoxylin and eosin (H.E.) staining. The lungs were fixed en block and subsequently cut into 1 mm-thick slices and embedded in 2% agar. Afterwards, the lung slices were paraffin-embedded and cut into 5 µm-thick sections. Ten sections of each paraffin wax block were H.E. stained and metastases were counted at a 200-fold magnification using Zeiss Axiophot photomicroscope (Zeiss, Jena, Germany). The total number of lung metastases was determined for all mice using quantitative assessment as described before (Jojovic and Schumacher 2000). Additionally, two series of serial sections out of the middle of each paraffin wax block were preserved for further immunohistological analyses.

### DNA extraction and real-time PCR for detection of circulating tumour cells, disseminated tumour cells, and lung metastases

DNA extraction of 200 µl murine blood was performed using the QIAamp DNA Blood Mini Kit (Qiagen, Hilden, Germany), and for DNA isolation of murine bone marrow and lung, the QIAamp DNA Mini Kit (Qiagen, Hilden, Germany) was used, according to manufacturer`s instructions. A serial dilution with tenfold dilution of extracted DNA from 1 × 10^6^ cell culture cells MDA MB231 to one cell was established. Control samples were isolated from mice without injected tumour cells. To quantify human tumour cells by real-time polymerase chain reaction (PCR), established primers specific for human Alu-sequences were used as described before (Nehmann et al. 2010).

Analyses were performed in triplicates and at least in two independent experiments, for each sample.

### RNA isolation and cDNA microarray analysis

Approximately 50 mg of fresh-frozen primary tumour tissue was crushed in liquid nitrogen. The total RNA was isolated using QIAzol Lysis Reagent (Qiagen, Hilden, Germany) and the miRNeasy Mini Kit (Qiagen, Hilden, Germany), according to manufacturer`s instruction. RNA yield was determined by UV absorbance using NanoDrop 1000 Spectrophotometer (Peqlab, Erlangen, Germany). The RNA quality was assessed by analysis of ribosomal RNA band integrity on an Agilent 2100 Bioanalyzer and RNA 6000 LabChip kit (Agilent Technologies, Palo Alto, CA, USA). The RIN values of RNA samples used for microarray analysis were higher than 7.7. The microarray experiments were performed according to the manufacturer's instructions (TermoFisher Scientific UserGuide P/N 703174) and as described in (Khaustova et al. 2017; Kudriaeva et al. 2017). Procedures for cDNA synthesis and labeling were carried out according to the GeneChip WT PLUS Reagent Kit (Applied Biosystems) protocol using 500 ng of total RNA as the starting material. Target DNA fragmentation, labeling, hybridization on Affymetrix Gene Chip Human Transcriptome Array 2.0 microarrays, array washing, staining, and scanning were performed as described in Sakharov et al. (2012). Background correction of the raw microarray data (CEL-files), normalization, and differential expression analysis were carried out using limma v3.13 R package (Ritchie et al. 2015). Functional annotation analysis was performed with DAVID v6.8 (Huang et al. 2009). All CEL-files are available in the Gene Expression Omnibus database (www.ncbi.nlm.nih.gov/geo/) under accession number GSE148089.

### qRT-PCR analysis

RNA was reverse transcribed to cDNA using 2 µg of total RNA as a starting material. Quantitative PCR analysis was carried out using the SYBR Green 5 × qPCRmix-HS SYBR reaction mix (Evrogen, Moscow, Russia). Amplification included 10 min denaturation at 94 °C, 40 cycles 20 s melting at 94 °C followed by 10 s annealing at 64 °C and 15 s elongation at 72 °C. Primer pairs were designed and characterized as described in Maltseva et al. (2013).

PCR efficiencies of all primer sets were higher than 1.9 and lower than 2.17 (Table 1), except for *CTSS* (1.79), *LGALS1* (1.86), and *SRGN* (1.88). All RNA samples were analysed in triplicate and averaged. Target genes were normalized to the reference genes *PTMA, RPS23, EEF1A1, ACTB*, and *GAPDH,* and data were processed based on the ∆∆Ct method (Livak and Schmittgen 2001). Reference gene selection and validation were performed using the approach described in Maltseva et al. (2013).

**Table 1:** Primer sequences of genes used for qRT-PCR

Gene symbol	Forward primer (5'→3')	Reverse primer (5'→3')	Efficiency	SD (E)
TSPAN8	TGAGCGCCACAGGGGAAAGT	CTCCATTGACCAAACCGCAGCA	1.99	0.06
TSPAN6	TGTACTCCACAGAGAGATGCAGACA	ACGAGAGAGGCAGTAGGCGAGA	1.95	0.16
DSC2	CTTGCATCAGACCAAGGAGGGAG	AGGCTCATCAGGATCAACCGCA	1.95	0.1
GALNT11	TGTCATAGACCGCACGCCAG	GCTGTCCAGGAACACAAGGACT	1.9	0.16
ADRM1	TTGCCATCTGGGGAGTCGCT	AAACGCTTCCACATCGCCCT	2	0.14
BABAM1	TTCGGACACCAAGGGTCAACTGT	ACGACTCCAGCTTTGGCAGTGA	1.96	0.08
ADGRF5 (GPR116)	CCTCATCCCTTCCTGCTGCAAAA	GACTTTCCCCGGCTCTCCGA	1.94	0.09
ESAM	GCTGGAAGTGAGCACAGGGC	GGAGCAATGGCATCCTCCTTGA	2.05	0.06
CTSS	CCCTGGATCACCACTGGCATCT	AGCACCACAAGAACCCATGTCTC	1.79	0.12
MGAT4A	AGCGGCAACCAAGAACATCCTG	CCACCATTCCTTCTGCAACACCA	2.04	0.09
LGALS1	ATCATGGCTTGTGGTCTGGT	GCACGAAGCTCTTAGCGTCA	1.86	0.11
MTA2	AGCAGAGTGCTCCATTCGAC	GGCCACCAGATCTTTGACGA	2.04	0.21
B4GALT6	ACGTATCTCCCGGAAAACTTCA	AATCCTCGCATATAAGGCAGC	1.99	0.15
CTTN	AGGTGTCCTCTGCCTACCAG	AGCCGCATCCTCATAGACG	1.95	0.09
SRGN	TCTGCAAACTGCCTTGAAGAAAA	CCTGGATTCTCGTCTTTGGAAAA	1.88	0.12
Reference genes				
PTMA	ACCAGCTCCGAAATCACCACCA	AGCAGGGGCGTCTCTTCCAT	2.17	0.08
RPS23	CCGTAGTCACCGACGAGACCA	TTGGCTGTTTGGCTTCAACTCCT	2.09	0.09
EEF1A1	CCCTAAAAGCCAAAATGGGAAA	TAGTGGTGGACTTGCCCGAAT	1.98	0.07
ACTB	CTGGAACGGTGAAGGTGACA	AAGGGACTTCCTGTAACAACGCA	2.03	0.08
GAPDH	GAAGGTGAAGGTCGGAGTC	GAAGATGGTGATGGGATTTC	2.02	0.12

### Protein extraction and Western blot analysis

For total protein extraction, about 100 mg fresh-frozen primary tumour tissue was crushed in liquid nitrogen and immediately suspended in ice cold self-made RIPA lysis buffer containing a protease inhibitor cocktail (Calbiochem, Darmstadt, Germany). The suspension was stored at − 20 °C until usage. Protein content of each sample was determined by BCA protein assay (Pierce BCA Protein Assay kit, Thermo Scientific, Rockford, IL, USA). Western blots were performed as described previously (Schroder et al. 2009). For validation of differentially expressed genes, the following primary and secondary antibodies are specified below; visualization by chemiluminescence reagents (SuperSignal West Pico Chemiluminescent Substrate kit, Thermo Scientific, Rockford, IL, USA) with Hyperfilm ECL films (GE-Healthcare, Freiburg, Germany). Band intensities were quantified by densitometry with GS-800 Calibrated Densitometer (BioRad, München, Germany). The intensities of the specific protein bands were calculated as percentage intensity of the control sample and corrected for equal actin loading.

### Commercial antibodies used for Western blot analysis

#### Primary antibodies

ß-Actin (RRID: AB_626632; cl C-4;1:500; Santa Cruz, Heidelberg, Germany).

Fra-2 (RRID: AB_2107084; cl Q-20;1:200; Santa Cruz, Heidelberg, Germany).

HCAM (RRID: AB 627,066; CD44; cl F-4; 1:200; Santa Cruz, Heidelberg, Germany).

ICAM-1 (RRID: AB_627123; cl G-5; 1:250; Santa Cruz, Heidelberg, Germany).

Integrin β4 (RRID: AB_626839; G-7; sc-13127; 1:500; Santa Cruz, Heidelberg, Germany).

L1-CAM (cl UJ 127.11; 1:25; obtained from Altevogt Lab, Heidelberg, (Ebeling et al. 1996)).

ALCAM (RRID: AB_868825; ab49496; 1:500; Abcam, Cambridge, United Kingdom).

#### Secondary antibodies

HRP goat anti-mouse IgG (RRID: AB_631736; 1:8000; sc-2055; Santa Cruz, Heidelberg, Germany).

HRP goat anti-rabbit IgG (RRID: AB_631748; 1:8000; sc-2054; Santa Cruz, Heidelberg, Germany).

### Immunohistochemistry

Immunohistochemical analyses were performed on paraffin-embedded sections of the primary tumour and right lungs. Sections were deparaffinized with xylene and rehydrated in a series of graded ethanol to distilled water. Primary Antibodies, secondary antibodies, and pretreatment are described below. All sections were counterstained with Mayer`s hemalum. Photographs were taken using Zeiss Axiophot 2 photomicroscope (Zeiss, Jena, Germany).

Commercial antibodies and pretreatment used for immunohistochemical analysis:

**Table:** 

Antibodies	Pretreatment
Fra-2 (RRID: AB_2107084; cl Q-20;1:50) (Santa Cruz, Heidelberg, Germany)	S1699 (DAKO), microwave
CD44 (RRID: AB_307182; cl L178; 1:100) (BD—Pharmingen, Heidelberg, Germany)	S1699 (DAKO), steamer, 121 °C
ICAM-1 (RRID: AB_627123; cl G-5; 1:500) (Santa Cruz, Heidelberg, Germany)	S1699 (DAKO), microwave
Integrin β4 (RRID: AB_10866385; cl 439-9B; 1:25) (Abcam, Cambridge, UK)	S1699 (DAKO), microwave
LGALS1 (RRID: AB_2136615; ab25138; 1:400) (Abcam, Cambridge, UK)	Citrate buffer, water bath, 99 °C
Cortactin (CTTN; RRID: AB_2088281; sc11408; 1:50) (Santa Cruz, Heidelberg, Germany)	S1699 (DAKO), microwave
Tspan8 (RRID: AB_2678899; HPA-044337; 1:50) (Atlas; Merck, Taufkirchen, Germany)	S1699 (DAKO), 125 °C
L1-CAM (RRID: AB_2133065; UJ127) (Abcam, Cambridge, United Kingdom)	EDTA, microwave
*Secondary antibodies* Anti-mouse IgG (RRID: AB_631736; sc-2005; 1:8000; Santa Cruz, Heidelberg, Germany) Anti-rabbit IgG (RRID: AB_631746; sc-2004; 1:8000; Santa Cruz, Heidelberg, Germany)	

### Statistical analyses

Data were visualized analysed using Graph Pad Prism 5.0 (GraphPad Software, La Jolla, CA) and SPSS 22.0 software (IBM, Chicago, IL, USA). An ANOVA with post hoc group comparisons model was used to compare tumour weight between control and Fra-2 cl 1 and 2. Due to the explorative nature, no adjustment for multiplicity was applied. For the number of lung metastases and comparison of disseminated tumour cells, a Negative Binomial Generalized Linear Model was applied. The potential confounders tumour weight and time of tumour growth were no independent factors and therefore excluded in all final models. Survival of mice was analysed by Kaplan–Meier survival curves and log-rank tests. All tests were performed at a significance level of *p* = 0.05. All p values are two-sided.

## Results

**Fig. 1:**
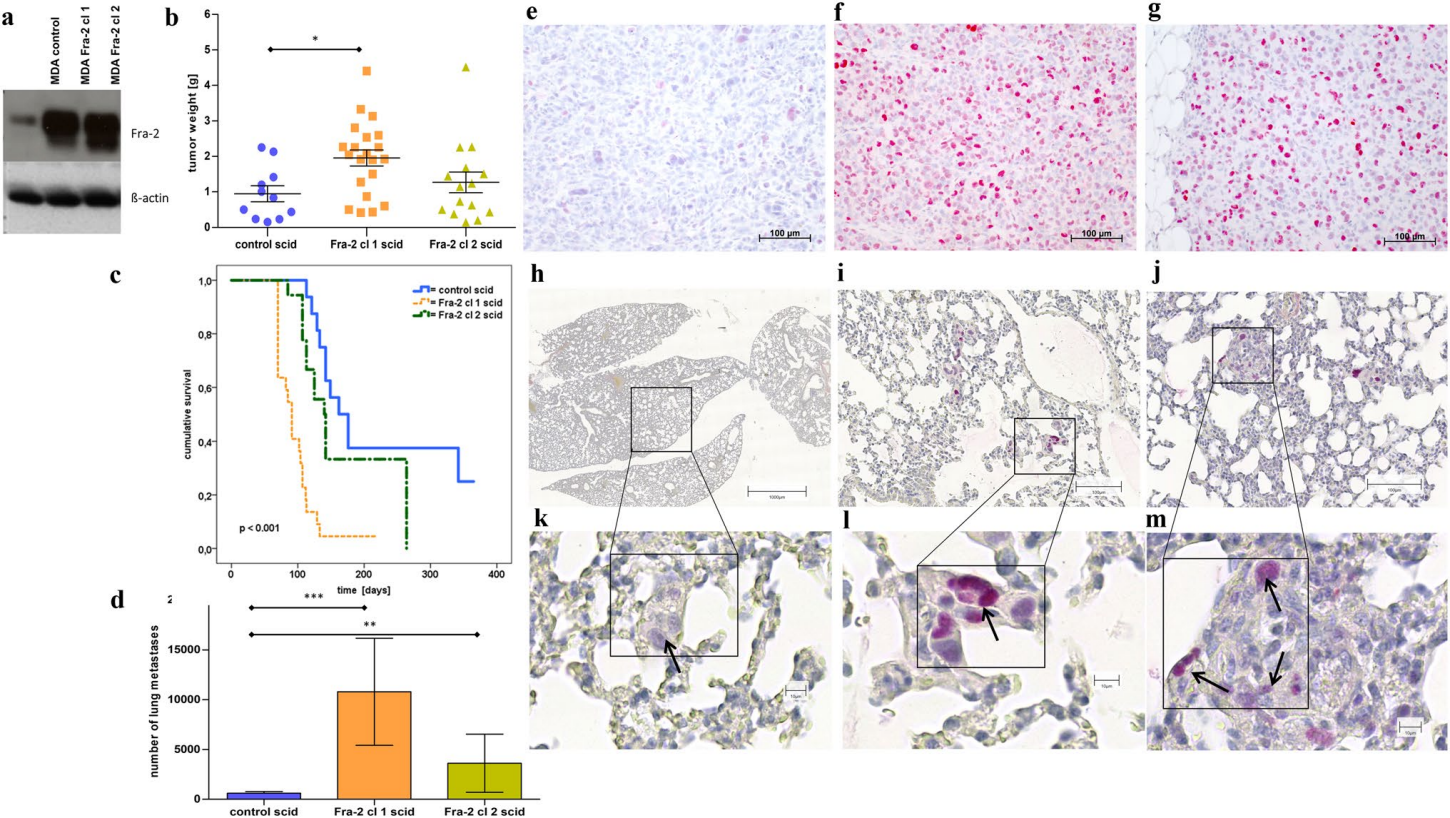
Effect of Fra-2 overexpression on MDA MB231 cells in vitro and in vivo. **a** Western Blot analysis of human total cell lysates showing Fra-2 overexpression in clones 1 and 2 relative to the MDA MB231 stably transfected cells harboring the empty pIRES-P vector (MDA control). ß-actin was used as loading control. The Blot is cropped for clarity, with full-length blot presented in Fig S1. **b** Tumour weight of the resected primary tumours from the scid mice. The tumour weight at the time of death was significantly smaller of the control primary tumours than Fra-2 cl 1 tumours (control mean tumour weight = 0.67 g, Fra-2 cl 1 mean tumour weight = 1.63 g, *p* = 0.018; Fra-2 cl 2 mean tumour weight = 0.87 g, *p* = 0.513) (***p < 0.005; **p < 0.01; *p < 0.05). **c** Kaplan–Meier survival curves of Fra-2 overexpressing human MDA MB231 cells transplanted subcutaneously into scid mice (blue line: scid mice transplanted with the empty vector pIRES = control; deepyellow line: scid mice with transplanted Fra-2 cl 1 cells; lightgreen line: scid mice with transplanted Fra-2 cl 2 cells (*p* < 0.001). Expression of the transcription factor Fra-2 dramatically decreases survival of the mice after subcutaneous injection of human breast cancer cells overexpressing Fra-2. **d** Number of microscopically detectable lung metastases in scid mice showing significant differences between Fra-2 overexpressing and control cells (***p < 0.005; **p < 0.01; *p < 0.05). Bars represent SEM. **e**–**m** Immunohistochemical staining of primary tumours and lung tissues with metastasized cells from scid mice; minimal Fra-2 expression in a primary tumour (**e**) and in metastasized cells of lung tissue (**h**, **k**) from a scid mouse transplanted with control cells; strong Fra-2 expression in a primary tumour and in metastasized cells of lung tissue from a scid mouse with transplanted Fra-2 cl 1 cells (**f**, **j** and **l**) and Fra-2 cl 2 cells (**g**, **j** and **m**). Arrow marks metastatic deposit. Scale bar: 100 and 10 µm

To analyse the functional role of Fra-2 in breast cancer cells, we established stable transfectants with overexpression of the transcription factor in the cell line MDA MB231 with the pIRES-Fra-2 vector as previously described (Schroder et al. 2010). Strongly enhanced Fra-2 expression levels could be confirmed in two single-cell clones (Fra-2 cl 1 and cl 2) by western blot analysis (Fig. 1a).

### Fra-2 overexpression leads to shortened overall survival and increased number of metastases in a subcutaneous scid xenograft model of human MDA MB231 cells

To investigate the effects of Fra-2 in vivo, cells of both clones with Fra-2 overexpression as well as control cells were injected subcutaneously into scid mice (Fra-2 cl 1: 22 mice; Fra-2 cl 2: 18 mice; control: 16 mice; total amount of 56 female mice) for a survival experiment. 47 of 56 mice developed primary tumours, particularly 95.5% of mice inoculated with Fra-2 cl 1, 83.3% of mice inoculated with Fra-2 cl 2 as well as 68.8% of mice which were injected with control cells. At necropsy excised control primary tumours were significantly smaller than Fra-2 cl 1 tumours, whereas the difference to cl 2 tumours did not reach statistical significance (control mean tumour weight = 0.67 g, Fra-2 cl 1 mean tumour weight = 1.63 g, Fra-2 cl 2 mean tumour weight = 0.87 g; control vs Fra-2 cl 1 *p* = 0.018; control vs Fra-2 cl 2 *p* = 0.513) (Fig. 1b). The time span between injections of the tumour cells until sacrifice will subsequently be referred to as survival time. Survival analysis demonstrated a significantly longer overall survival of the mice injected with control clone compared to mice injected with the Fra-2 clones 1 and 2 (range 70–342 days, median survival control = 162 days, median survival Fra-2 cl 1 = 91 days, and median survival Fra-2 cl 2 = 140 days, *p* < 0.001) (Fig. 1c). The presence of microscopically detectable lung metastases was confirmed upon histological examination of the right lung. 94.1% of mice injected with Fra-2 cl 1 developed lung metastases (range from 264 to 93,746 lung metastases, mean = 10,785). We detected lung metastases in 54.5% of mice injected with Fra-2 cl 2 cells (range from 140 to 29,744 lung metastases, mean = 3295, whereas significantly fewer metastases were found in the control group injected with control cells (lung metastases were found in 84.6% of control mice, range from 89 to 1766, mean = 576; control vs Fra-2 cl 1 *p* < 0.001, control vs Fra-2 cl 2 *p* = 0.007) (Fig. 1 d).

To demonstrate that the Fra-2 overexpression still existed in primary tumours grown in scid mice and their metastases, we analysed the Fra-2 expression by immunohistochemistry. Elevated Fra-2 protein levels could be detected in primary tumours of Fra-2 scid mice (Fig. 1e–g) and could also be observed in corresponding lung metastases (Fig. 1 h–m).

### Microarray analyses of resected tumours and validation of genes with dysregulated expression in scid mouse tumours

Since Fra-2 overexpression also resulted in an increased number of metastases and reduced overall survival in scid mice, cDNA arrays with mRNA isolated from xenograft tumour tissue were performed and analysed (three tumours per group). Genes that were at least twofold increased or decreased compared with the respective control were further considered to be significantly dysregulated (adjusted *p* value below 0.05). According to these criteria, the expression of 63 genes were altered in scid primary tumours of both clones at the same time (upregulated: 27 genes, downregulated: 36 genes). The further enrichment analysis showed that alterations of extracellular matrix and cell surface were the most prominent function attributed to these genes followed by “signal peptide” and “calcium ion binding”. The analysis of two clones separately revealed 65 (control vs Fra-2 cl 1) and 33 (control vs Fra-2 cl 2) genes, respectively, that were upregulated and 47 (control vs Fra-2 cl 1) and 45 (control vs Fra-2 cl 2) genes, respectively, that were downregulated in scid primary tumours.

**Fig. 2:**
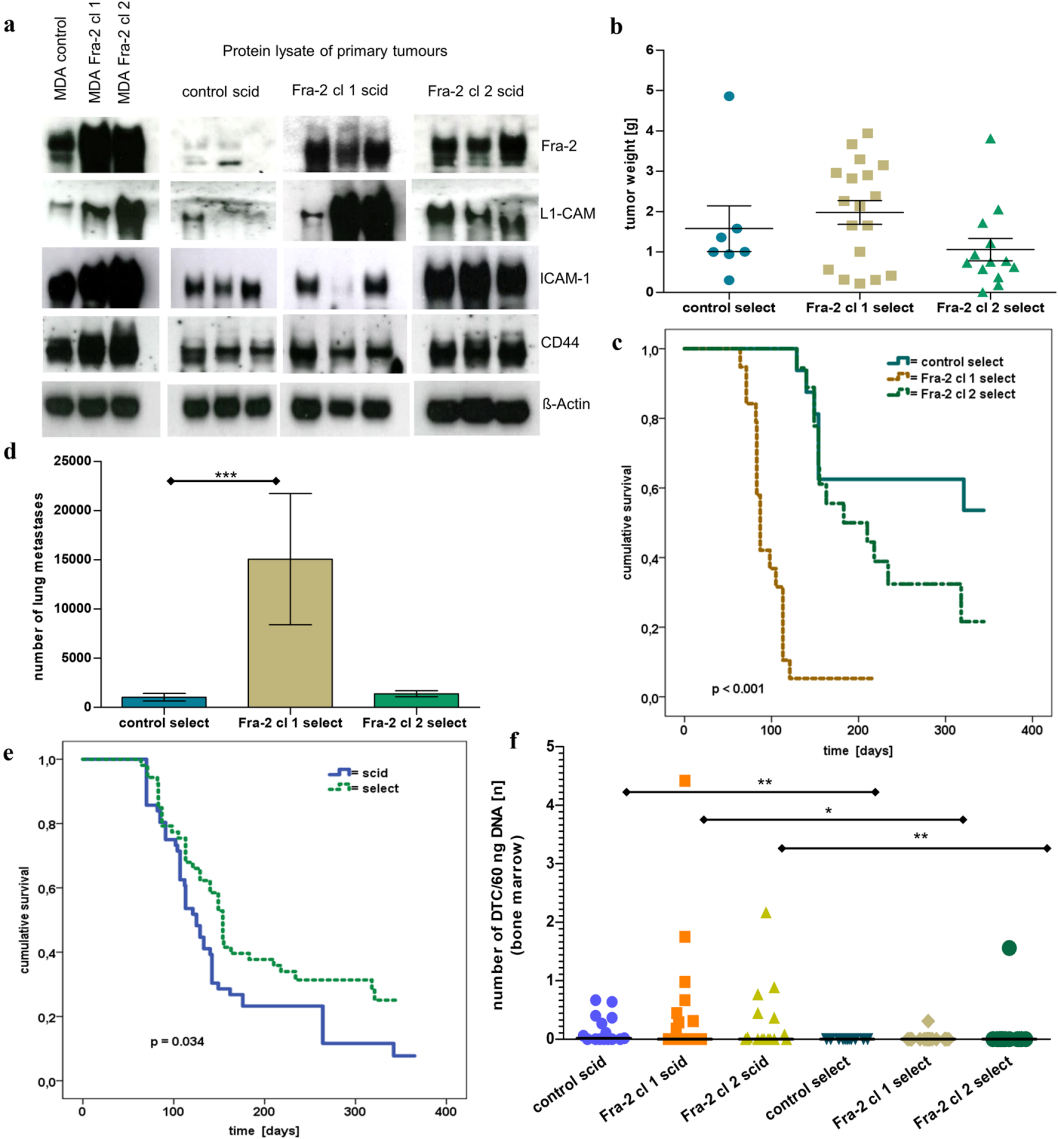
**a** Western blot analysis; cell lysates of the transfected MDA MB231 cells (first 3 rows) and protein lysates of the resected scid mouse primary tumours of control (second 3 rows), Fra-2 cl 1 and 2 cells with staining of L1-CAM, ICAM-1, CD44; (loading control ß-actin). Original non-cropped images of the scanned immunoblot membranes are shown in Figs S2 (**a**–**d**), respectively. **b** Tumour weight of the resected primary tumours from the select mice. No significant difference between the tumour weights of control and both Fra-2 clones in the select mice (mean tumour weight: control = 1.17 g, Fra-2 cl 1 = 1.43 g, *p* = 0.656; Fra-2 cl 2 = 0.61 g, *p* = 0.165) (***p < 0.005; **p < 0.01; *p < 0.05). **c** Kaplan–Meier survival curves of Fra-2 overexpressing human MDA MB231 cells transplanted subcutaneously into select mice revealed significantly longer overall survival of the select mice injected with control cells compared with select mice injected with the Fra-2 cls 1 and 2 cells, similar to the results in scid mice (range 64,321 days, median survival: Fra-2 cl 1 = 87 days, and Fra2 cl 2 = 183 days, *p* < 0.001) (oceanblue line: select mice with the empty vector pIRES = control; yellowocher dotted line: select mice with Fra-2 cl 1 cells; mossgreen dashed line: select mice with Fra-2 cl 2 cells (*p* < 0.001). **d** Number of microscopically detectable lung metastases in select mice; the difference between control and Fra-2 cl 1 is clearly significant (*p* < 0.005), respectively (***p < 0.005; **p < 0.01; *p < 0.05). **e** Remarkably, comparing by Kaplan–Meier analysis, survival curves of scid (blue continuous line) and select mice (green dotted line) showing a significantly better prognosis for the select mice (median survival: scid = 125 days, select = 154 days, *p* = 0.034). **f** DTCs in the animals´ bone marrow showing a significantly reduced number of disseminated tumour cells in the select mice compared to the scid mice (*p* = 0.014) (***p < 0.005; **p < 0.01; *p < 0.05). Bars represent SEM

**Fig. 3:**
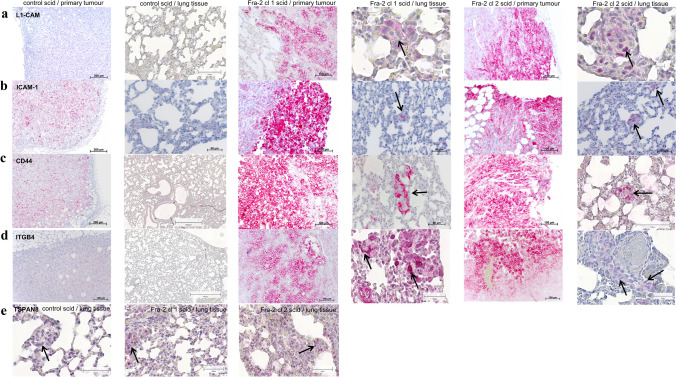
Immunohistochemistry of primary tumours engrafted in scid mice and their spontaneous metastases in the lung tissue. **a**–**d** IHC staining of representative paraffin-embedded primary tumour tissue and lung tissue with metastasized cells grown in scid mice showing a higher immunoreactivity of L1-CAM, ICAM-1, CD44, and ITGB4 in the tumour cells. Scale bar: 200 and 50 µm. **e** Images of paraffin-embedded lung sections of scid mice display an immunoreactivity of TSPAN8 within the metastasized cells. This could indicate that these genes are essential for the tumour cells during the emigration process. Arrows label metastasized cells in the lungs. Scale bar: 100 µm

The focus of our analyses was directed on genes that are commonly associated with altered adhesion behavior of tumour cells and/or carrying selectin-binding sites on the cell surface. In the analysed groups, no concordant genes were identified in the resected tumours of injected Fra-2 cl 1 and Fra-2 cl 2 cells, but prospective candidates with corresponding characteristics were found among the upregulated genes in both Fra-2 groups. In particular, genes such as *L1-CAM, ITGB4, DSC2* (Desmocollin 2), *TSPAN8* (Tetraspanin 8), or *GPR116* have been upregulated and *MAN1A1* or *MMP1* have been downregulated compared to the control (Table 2). Using qRT-PCR, we could validate Fra-2-mediated upregulation for AGRF5 (GPR116), TSPAN8, DSC2, TSPAN6, and MGAT4A (Table 3). We chose L1-CAM, ICAM-1, and CD44—based on earlier results (Schroder et al. 2010)—and ITGB4 and TSPAN8 to exemplarily verify the microarray results on protein level. In a western blot analysis, we were able to confirm a different regulation of L1-CAM and CD44 in both clones compared to the respective control samples using protein lysates obtained from tumours of the resected mice (Fig. 2a) However, we could only detect a higher expression of ICAM-1 in Fra-2 cl 2 (Fig. 2a), but by immunohistochemical staining of sections from primary tumours and metastases of lung tissue, we could validate expression differences of ICAM-1 between both Fra-2 clones compared to the control (Fig. 3b). We also found a visibly stronger expression of L1-CAM, CD44, and ITGB4 in the scid mice primary tumours and lung metastases injected with Fra-2 overexpressing cells by immunohistochemical staining (Fig. 3a,c,d). For TSPAN8, however, we could only detect a positive immunoreactivity in the lungs (Fig. 3e).

**Table 2:** Selection of genes, which were deregulated by Fra-2 overexpression in scid mice

Gene symbol	Gene title	Fold change	*p* value	Validation method	Fold Change	*p* value
	Control scid vs Fra-2 cl 1 scid tumour Increase				Control scid vs Fra-2 cl 2 scid tumour	
*GPR116*	G protein-coupled receptor 116	2.0	0.00208763	qRT-PCR	2.6	0.02192253
*TSPAN8*	Tetraspanin 8	2.1	0.002	qRT-PCR	1.5	0.0785871
*ITGB4*	Integrin, beta 4	1.5	0.00033547	IHC	1.1	0.72106998
*DSC2*	Desmocollin 2	2.1	0.00006535	qRT-PCR	2.2	0.05738855
*L1-CAM*	L1 cell adhesion molecule	1.4	0.01070848	WB/IHC	2.0	0.21294704
*ITGA10*	Integrin, alpha 10; integrin alpha-10-like	1.5	0.0015988	n/a	1.2	0.62095434
	Control scid vs Fra-2 cl 1 scid tumour Decrease				Control scid vs Fra-2 cl 2 scid tumour	
*MAN1A1*	Mannosidase, alpha, class 1A, member 1	− 5.4	0.00019338	n/a	− 2.4	0.01352611
*SRPX*	Sushi-repeat containing protein, X-linked	− 2.0	0.0052303	n/a	− 1.7	0.11159797
*TNC*	Tenascin C	− 2.2	0.00055037	n/a	− 1.4	0.41584994
*CTSL1*	Cathepsin L1	− 2.1	0.00008895	n/a	− 1.6	0.06486182
*CXCR4*	Chemokine (C-X-C motif) receptor 4	− 2.1	0.00030803	n/a	− 1.8	0.01434181
*MAN2A1*	Mannosidase, alpha, class 2A, member 1	− 1.5	0.01363244	n/a	− 1.2	0.1078938
*ITGB8*	Integrin, beta 8	− 1.7	0.00027035	n/a	− 1.7	0.00171451
*ALCAM*	Activated leukocyte cell adhesion molecule	− 1.7	0.00766435	IHC not shown	− 1.3	0.09498473
	Control scid vs Fra-2 cl 2 scid tumour Increase				Control scid vs Fra-2 cl 1 scid tumour	
*CTSS*	Cathepsin S	2.1	0.00068139	qRT-PCR	1.3	0.04249498
*TSPAN6*	Tetraspanin 6	1.6	0.00198598	qRT-PCR	1.5	0.03193598
*MGAT4A*	Mannosyl (alpha-1,3-)-glycoprotein beta-1,4-*N*-acetylglucosaminyltransferase, isozyme A	1.8	0.00238961	qRT_PCR	1.2	0.10289642
	Control scid vs Fra-2 cl2 scid tumour Decrease				Control scid vs Fra-2 cl 1 scid tumour	
*MMP1*	Matrix metallopeptidase 1	− 4.3	0.0010812	IHC not shown	− 2.4	0.00400168
*ST8SIA4*	ST8 alpha-*N*-acetyl-neuraminide alpha-2,8-sialyltransferase 4	− 1.7	0.01635993	qRT_PCR not shown	− 1.5	0.03548038
*B4GALT5*	UDP-Gal:betaGlcNAc beta-1,4-galactosyltransferase, polypeptide 5	− 1.5	0.03930351	qRT_PCR not shown	− 1.2	0.16447326
*ITGB8*	Integrin, beta 8	− 1.7	0.00171451	n/a	− 1.7	0.00027035
*CSGALNACT2*	Chondroitin sulfate *N*-acetylgalactosaminyltransferase 2L	− 1.5	0.00291929	n/a	− 1.4	0.01102847

The entire list of data is deposited in the Gene Expression Omnibus database (see Data availability)

**Table 3:** Validation of microarray results by qRT-PCR of selected genes

Gene symbol	Fra-2 cl2 vs pIRES		Fra-2 cl8 vs pIRES		Fra-2 (cl2+cl8) vs pIRES	
	FC	*p* value	FC	*p* value	FC	*p* value
AGRF5 (GPR116)	5.2	< 0.00001	13.0	0.009	9.1	0.014
TSPAN8	7.4	0.00014	2.8	0.009	5.1	0.002
DSC2	3.1	< 0.00001	1.8	*0.16*	2.5	0.015
CTSS	1.2	*0.18*	2.1	0.007	1.7	0.033
TSPAN6	1.6	0.0008	3.5	0.000016	2.5	0.0014
MGAT4A	2.0	0.00001	8.3	0.003	5.2	0.019

Data in italics are not statistically significant

Table 2 is included in a separate file.

### E- and P-selectin deficiency in mice leads to longer overall survival

To examine whether E- and P-selectin and tumour cell interactions took part in malignant progression in vivo, the transfected cells were injected subcutaneously in a total amount of 53 E- and P-selectin double knock-out scid mice (select), 19 of them with Fra-2 cl 1 cells, 18 with Fra-2 cl 2 cells, and 16 with control cells. Primary tumour growth was observed in 38 of 53 mice (71.7%), more precisely in 18 of 19 mice injected with Fra-2 cl 1 cells (94.7%) and in 13 of 18 mice injected with Fra-2 cl 2 cells (72.2%), whereas only 7 of 16 (43.8%) of the control injected mice developed primary tumours after a time period of 300 days in the survival experiment. There was no significant difference between the tumour weights of controls and both Fra-2 clones in the select mice (control mean tumour weight = 1.17 g, Fra-2 cl 1 mean tumour weight = 1.43 g, Fra-2 cl 2 mean tumour weight = 0.61 g; control vs Fra-2 cl 1 *p* = 0.656; control vs Fra-2 cl 1 *p* = 0.165) (Fig. 2b), although the termination criteria between the different groups were applied in the same way. However, survival analysis demonstrated a significantly longer overall survival of the select mice injected with control cells compared with select mice injected with the Fra-2 cls 1 and 2 cells, similar to the results in scid mice (range 64–321 days, median survival Fra-2 cl 1 = 87 days, median survival Fra2 cl 2 = 183 days, *p* < 0.001) (Fig. 2c).

Spontaneous lung metastases were histologically detected in 50% of Fra-2 control cells in select mice (range from 560 to 2786 lung metastases, mean = 620). In comparison, lung metastases were found in 100% of select mice injected with Fra-2 cl 1 cells (range from 1591 to 107,184 lung metastases, mean = 15,074; *p* < 0.001), while in 66.7% of select mice injected with Fra-2 cl 2 cells, microscopically detectable lung metastases were found (range from 414 to 2688 lung metastases, mean = 1115; *p* = 0.155) (Fig. 2d).

On comparison of the two experiments to estimate the survival function between the scid and select mice, a significantly shorter overall survival of scid (*n* = 56) mice versus select (*n* = 53) mice could be observed (median survival scid = 125 days, median survival select = 154 days, *p* = 0.034) (Fig. 2e). Due to the median survival advantage of almost 29 days, we assumed that the selectin status of the immunodeficient mice did directly influence overall survival.

Remarkably quantitative real-time Alu-PCR of bone marrow did show significantly reduced numbers of disseminated tumour cells in the select mice compared to the scid mice (mean of DTCs scid mice = 0.2430 cells/60 ng template DNA, mean of DTCs select mice = 0.0360 cells/60 ng template DNA, *p* = 0.014) (Fig. 2f). Almost no detectable human cells were found in the select mice except in one injected with Fra-2 cl 1 cells (0.31 cells/60 ng template DNA) and one injected with Fra-2 cl 2 cells (1.56 cells/60 ng template DNA) (4.3% of mice) compared to the scid mice, where in 47.1% of mice, human DNA was detected in the bone marrow. Taken together, our data suggest an essential effect of the selectins on cell engraftment in the bone marrow and lungs.

### Microarray analyses of resected tumours and validation of genes with dysregulated expression in select mice tumours

As Fra-2 overexpression with Fra-2 cl 1 cells transplanted into select mice also led to an increased metastatic load and significantly reduced survival in the select mice; again, microarray analyses were performed as described before.

According to our criteria, 369 genes were differentially expressed in select primary tumours of both clones at the same time (upregulated: 173 genes, downregulated: 196 genes). The further enrichment analysis showed that alterations of “membrane”, “extracellular matrix”, “extracellular exosome”, “cell adhesion”, and “focal adhesion” were the most prominent among the affected molecular functions’ groups. The analysis of two clones separately revealed 146 (control vs Fra-2 cl 1) and 457 (control vs Fra-2 cl 2) upregulated genes, respectively, and 258 (control vs Fra-2 cl 1) and 328 (control vs Fra-2 cl 2) downregulated genes, respectively, in select mouse tumours, respectively.

Again, we focused on genes that could putatively play a role in the adhesion of tumour cells to endothelial cells as the gene product can interact with selectins. In both groups (Fra-2 cl 1 and cl 2), some interesting genes could be identified, e.g., in the Fra-2 cl 1 primary tumours *LGALS1, ADRM1,* and *CTTN*, which were upregulated and *LUM* (Lumican), *MAN1A1,* and *SPARC*, which were significantly downregulated. For Fra-2 cl 2 primary tumours, genes such as *SRGN* (Serglycin) and *ESAM* were upregulated, while *CD99* and *SELPLG* (selectin P ligand) were downregulated (Table 4).

**Table 4:** Selection of genes, which were deregulated by Fra-2 overexpression in select mice

Gene symbol	Gene title	Fold Change	ANOVA *p* value	Validation method	Fold change	ANOVA *p* value
	Control select vs Fra-2 cl 1 select tumour Increase				Control select vs Fra-2 cl 2 select tumour	
*LGALS1*	Lectin, galactoside-binding, soluble, 1	2.2	0.00431535	qRT-PCR/IHC	1.7	0.01798879
*MTA2*	Metastasis associated 1 family, member 2	1.8	0.00647685	qRT-PCR/IHC	1.5	0.05890602
*B4GALT6*	UDP-Gal:betaGlcNAc beta-1,4-galactosyltransferase, polypeptide 6	1.4	0.2966968	qRT-PCR	1.7	0.04574728
*ADRM1*	Adhesion regulating molecule 1	1.5	0.03346412	qRT-PCR	1.5	0.09002897
*CTTN*	Cortactin	1.5	0.0072002	qRT-PCR/WB	1.6	0.0200769
*GALNT11*	UDP-*N*-acetyl-alpha-d-galactosamine:polypeptide *N*-acetylgalactosaminyltransferase 11 (GalNAc-T11)	1.3	0.11281676	qRT-PCR	1.0	0.89901574
*BABAM1*	BRISC and BRCA1 A complex member 1	1.3	0.04428669	qRT-PCR	1.1	0.52440179
	Control select vs Fra-2 cl 1 select tumour Decrease				Control select vs Fra-2 cl 2 select tumour	
*CTSO*	Cathepsin O	− 3.6	0.00213909	n/a	− 4.2	0.00607428
*MGAT5*	Mannosyl (alpha-1,6-)-glycoprotein beta-1,6-*N*-acetyl-glucosaminyltransferase	− 3.2	9.65E-05	n/a	− 1.6	0.04360072
*MAN1A1*	Mannosidase, alpha, class 1A, member 1	− 4.7	0.00842035	n/a	− 3.8	0.012972
*MMP2*	Matrix metallopeptidase 2	− 1.8	0.20919243	IHC not shown	− 2.7	0.00117561
*CTSL1*	Cathepsin L1	− 2.1	0.01190166	n/a	− 1.8	0.01014816
*LUM*	Lumican	− 2.4	0.05014903	n/a	− 1.8	0.2663245
*PDGFC*	Platelet-derived growth factor C	− 2.0	0.00712603	n/a	− 2.7	0.04867148
*MANBA*	Mannosidase, beta A	− 2.3	0.00057998	n/a	− 3.0	0.0012044
*LDOC1*	Leucine zipper, downregulated in cancer 1	− 1.2	0.46218117	n/a	− 1.4	0.2096031
*SPARC*	Secreted protein, acidic, cysteine-rich (osteonectin)	− 1.5	0.0091225	n/a	− 1.8	0.15875616
*GALNT1*	UDP-*N*-acetyl-alpha-d-galactosamine:polypeptide *N*-acetylgalactosaminyltransferase 1 (GalNAc-T1)	− 1.5	0.03394277	n/a	− 2.0	0.15452444
*ZEB2*	ZEB2	− 1.7	0.00178154	n/a	− 1.7	0.01131962
*GALNT7*	UDP-N-acetyl-alpha-d-galactosamine:polypeptide *N*-acetylgalactosaminyltransferase 7 (GalNAc-T7)	− 1.5	0.06684931	n/a	− 1.4	0.30001461
*HAPLN3*	Hyaluronan and proteoglycan link protein 3	− 1.3	0.08629049	n/a	− 1.5	0.05355493
*SELPLG*	Selectin P ligand	− 1.5	0.01487063	IHC not shown	− 1.6	0.04884421
*ST6GALNAC5*	ST6 (alpha-*N*-acetyl-neuraminyl-2,3-beta-galactosyl-1,3)-*N*-acetylgalactosaminide alpha-2,6-sialyltransferase 5	− 1.2	0.08559022	n/a	− 1.1	0.50882932

Gene symbol	Gene title	Fold change	*p* value	Validation method	Fold Change	*p* value	
	Control select vs Fra-2 cl 2 select tumour Increase				Control select vs Fra-2 cl 1 select tumour		
*SRGN*	Serglycin	9.9	0.0000102	qRT-PCR	2.1	0.04986751	
*CTTN*	Cortactin	1.6	0.0200769	qRT-PCR/IHC	1.5	0.0072002	
*ESAM*	Endothelial cell adhesion molecule	1.4	0.0901989	qRT-PCR/IHC	1.5	0.01365101	
	Control select vs Fra-2 cl 2 select tumour Decrease				Control select vs Fra-2 cl 1 select tumour		
*CDON*	Cell adhesion associated, oncogene regulated	− 3.4	0.00022592	n/a	− 1.6		0.24959834
*MMP2*	Matrix metallopeptidase 2	− 2.7	0.00117561	IHC not shown	− 1.8		0.20919243
*CD99*	CD99 molecule	− 3.2	0.00014262	n/a	− 1.3		0.02444132
*MANBA*	Mannosidase, beta A	− 3.0	0.0012044	n/a	− 2.3		0.00057998
*PLAUR*	Plasminogen activator, urokinase receptor	− 2.7	0.00067957	n/a	− 1.8		0.17676286
*CTSB*	Cathepsin B	− 2.1	0.0021906	n/a	− 1.4		0.04344705
*DSG2*	Desmoglein 2	− 2.1	0.00965429	n/a	− 1.4		0.03179364
*OGT*	*O*-linked *N*-acetylglucosamine (GlcNAc) transferase	− 2.2	0.00615686	n/a	− 1.8		0.00149708
*SMAD4*	SMAD family member 4	− 2.0	0.00733559	n/a	− 1.2		0.09374416
*SMAD2*	SMAD family member 2L	− 2.0	0.01306913	n/a	− 1.3		0.05717904
*CTSL1*	Cathepsin L1	− 1.8	0.01014816	n/a	− 2.1		0.01190166
*GLB1*	Galactosidase, beta-1	− 1.7	0.01506378	n/a	− 1.3		0.07456839
*ST8SIA4*	ST8 alpha-*N*-acetyl-neuraminide alpha-2,8-sialyltransferase 4	− 1.9	0.00717723	n/a	− 1.2		0.67029747
*GJB2*	Gap junction protein, beta 2	− 2.3	0.00184347	n/a	− 1.2		0.44925495
*SELPLG*	Selectin P ligand	− 1.6	0.04884421	IHC not shown	− 1.5		0.01487063
*MAN2B2*	Mannosidase, alpha, class 2B, member 2	− 1.6	0.02517537	n/a	− 1.1		0.40962084
*ZEB2*	ZEB2	− 1.7	0.01131962	n/a	− 1.7		0.00178154
*SMAGP*	Small cell adhesion glycoprotein	− 1.5	0.04141548	n/a	− 1.0		0.87646386
*JUNB*	Jun B proto-oncogene	− 1.5	0.03292006	IHC not shown	− 1.0		0.93067704

The entire list of data is deposited in the Gene Expression Omnibus database (see Data availability)

**Fig. 4:**
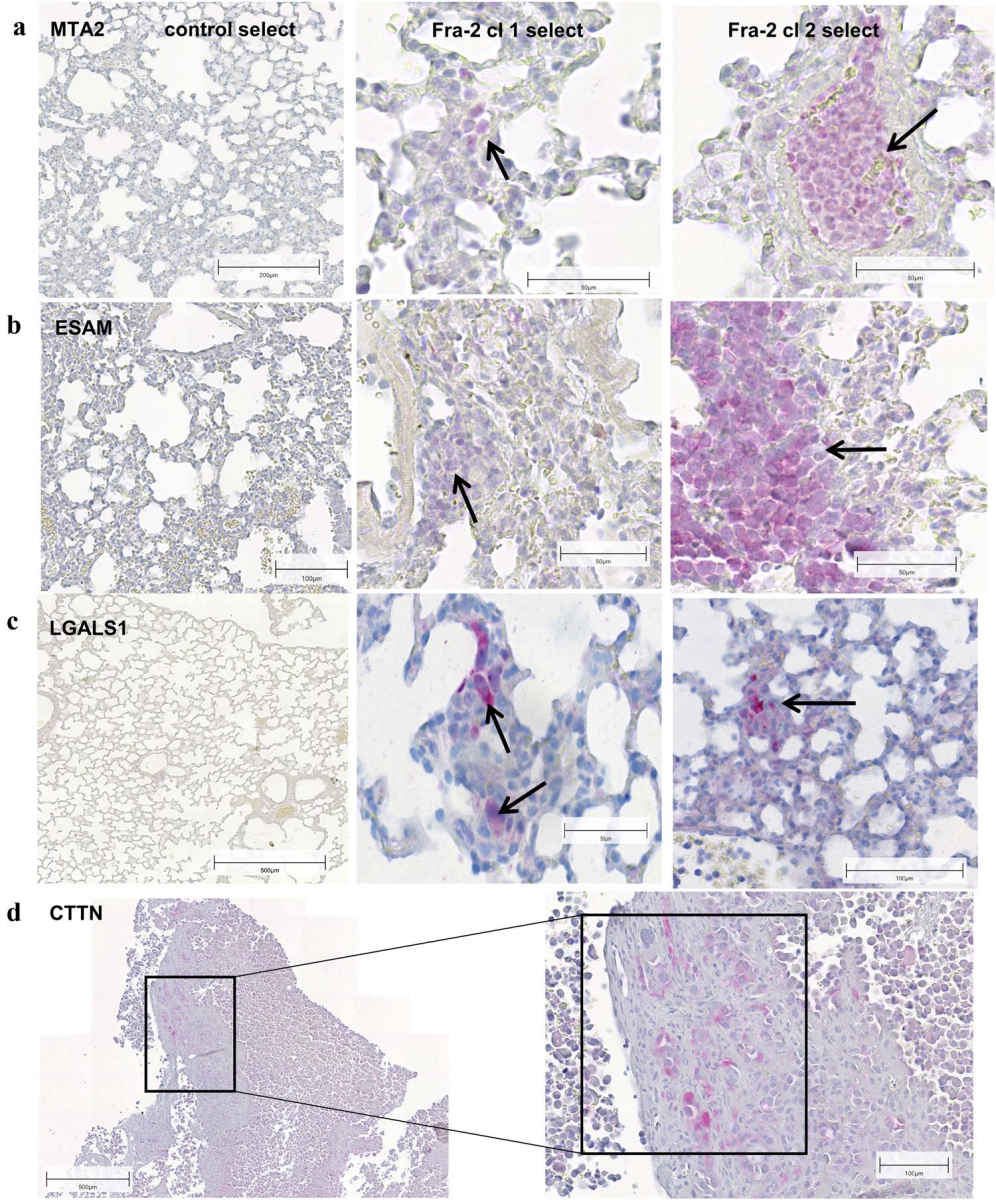
**a**–**d** IHC staining of selected upregulated proteins in representative lung tissue grown in select mice injected with Fra-2 clones showing a higher immunoreactivity of MTA2 (p <), ESAM (**b**), and LGALS1 (Galectin-1) (**c**) in the metastasized cells. Scale bar: 200—50 µm. Arrows label metastasized cells in the lungs. **d** IHC staining of primary tumour resected from select mice injected with Fra-2 clone 2 with CTTN-positive tumour cells. Scale bar: 500–100 µm

In addition, comparing Fra-2 cl 1 and cl 2 primary tumours in the select group, we found matched genes that were equally regulated by Fra-2 in both clones. *CTTN* was upregulated; meanwhile, *MMP2, ZEB2,* and *SELPLG* were downregulated in both clones. Using qRT-PCR, we could validate Fra-2 mediated upregulation for *ADRM1*, *GALNT11, BABAM1, ESAM, LGALS1,* and *CTTN* (Table 5). By immunohistochemical staining, we could confirm some of these gene products like MTA2, ESAM, and LGALS1 in primary tumour and in metastasized cells in the lung of the two clones (Fig. 4a–c), but CTTN only in primary tumour sections not in metastatic cells of the lung (Fig. 4d).

**Table 5:** Validation of microarray results by qRT-PCR of selected genes

Gene symbol	Fra-2 cl2 vs pIRES		Fra-2 cl8 vs pIRES		Fra-2 (cl2+cl8) vs pIRES	
	FC	*p* value	FC	*p* value	FC	*p* value
ADRM1	0.91	*0.24*	0.78	*0.11*	0.85	*0.15*
GALNT11	4.23	< 0.00001	1.38	0.049	2.81	0.028
BABAM1	1.48	0.000069	1.09	*0.40*	1.28	*0.14*
ESAM	8.32	< 0.00001	3.76	0.0041	6.04	0.0022
LGALS1	1.5	0.020	2.2	0.044	1.8	*0.051*
MTA2	1.9	*0.14*	1.1	*0.25*	1.5	*0.16*
B4GALT6	1.0	*0.45*	1.4	*0.12*	1.2	*0.20*
CTTN	4.7	0.0024	1.8	0.025	3.3	0.031
SRGN	1.8	*0.097*	13.6	0.00024	7.7	*0.053*

Data in italics are not statistically significant

Further evaluating the microarray data of the scid and the select group, we observed that many genes which are crucial for glycosylation show an altered expression: for example, *MAN1A1, MAN2A1, and GALNT 5* and *7* were downregulated in Fra-2 cl 1 scid primary tumours, whereas *MGAT4A* was upregulated. *ST8SIA4, B4GALT5,* and *CSGALNACT2* were found to be downregulated in Fra-2 cl 2 scid primary tumours.

*LGALS1, B4GALT6, and GALNT11* were upregulated and *MGAT5, MAN1A1, MANBA, LUM, GALNT1* and *7, HAPLN3,* and *ST6GALNAC5* were downregulated in Fra-2 cl 1 select primary tumours, while *MGAT4A* was upregulated.

Table 4 is included in a separate file.

## Discussion

The aim of the present experimental study was to elucidate the functional consequences of Fra-2 overexpression for the formation of metastases in a spontaneous breast cancer metastasis model. So far, only clinical and in vitro data were available, which showed that Fra-2 overexpression is associated with a significantly shorter disease-free survival for breast cancer patients and in Fra-2 overexpressing cell lines Fra-2 dysregulates genes involved in cell–cell and cell–matrix contacts (Schroder et al. 2010; Milde-Langosch et al. 2008).

Our results from the present scid mouse models demonstrate that Fra-2 overexpression in the injected breast cancer cells leads to a significantly shorter overall survival of the mice, and in accordance with this observation, a considerable and significantly higher number of microscopically detectable metastasis was detected in the animals´ lungs (Fig. 1e–m). To identify the genes involved in this enhanced metastatic capacity, cDNA microarray analysis of resected primary scid mouse xenograft tumours was performed which identified a considerable number of dysregulated genes, which are known to be involved in metastasis formation.

The attachment of the tumour cells to endothelium is the crucial step during metastasis formation, which is characterized by enhanced adhesive properties of the circulating cells by upregulation of cell-adhesion molecules and their ligands, respectively. The general steps of the metastatic cascade can be divided into several phases. The extravasation of tumour cells is initialized by rolling and low-affinity binding at the endothelium mediated by the selectin family. Rolling is followed by tight adhesion through integrins or other members of the large family of adhesion molecules. As cell-to-cell and cell-to-matrix adhesion molecules had been identified to be rate limiting for metastasis formation, we focused on this class of molecules (Gebauer et al. 2013; Kohler et al. 2010; Oliveira-Ferrer et al. 2015; Oliveira-Ferrer et al. 2014). Supporting our former in vitro study (Schroder et al. 2010), we found a higher mRNA expression of the immunoglobulin superfamily cell-adhesion molecule L1-CAM in isolated Fra-2 cl 1 scid primary tumours, which could be confirmed by Western blot (Fig. 2a) and IHC analysis including primary tumour sections and metastasized cells in the lungs (Fig. 3a). The expression of L1-CAM is induced by ERK–MAP-kinase signaling (Schaefer et al. 1999; Silletti et al. 2004) and MAP/ERK has been shown to be an important upstream regulator of AP-1 signaling (Eferl and Wagner 2003). Mechanistically, Fra-2, as heterodimer with a Jun partner, binds conserved AP-1 consensus sites at the L1-CAM promoter and is able to mediate transcriptional activation of L1-CAM when Fra-2 is phosphorylated by ERK-1/2 (Geismann et al. 2009). Results from Dippel et al. indicate that L1-CAM expression on MDA MB231 cells promotes adherence to activated endothelial cells by binding to endothelial L1-CAM (Dippel et al. 2013). Enhanced expression resulted in L1-CAM mediated cell adhesion, migration, and proliferation, thereby contributing to tumour progression and invasiveness. In nearly all malignancies investigated so far, L1-CAM expression was associated with poor prognosis, tumour progression, and metastasis to lymph nodes (Altevogt et al. 2016). A key player in this step of the metastatic cascade is ICAM-1, of which we confirmed a higher expression in the isolated Fra-2 cl 2 scid primary tumours by Western blot (Fig. 2a) and IHC analysis (Fig. 3b). ICAM-1 mediates the adhesion of lymphocytes to endothelial cells (Lawson and Wolf 2009) via Integrin αLβ2 (LFA-1) (Simon et al. 2000). As tumour cells do not express αLβ2 integrins, they use leukocytes as linker cells to adhere to the vascular endothelium by means of an ICAM-1/LFA-1 interaction (Strell and Entschladen 2008). It has been shown that MDA MB468 breast cancer cells use this mechanism to adhere to lung endothelial cells (Strell et al. 2007). Furthermore, ICAM-1 expression correlated with the metastatic capacity of five human breast cancer cell lines, suggesting its key role in invasion and dissemination (Rosette et al. 2005). As ICAM-1 contain AP-1 binding sites within their promoter regions, ICAM-1 engagement leads to downstream activation of the MAP-and ERK-1 kinase cascade and subsequent AP-1 transcription factor activity (Hubbard and Rothlein 2000; Lawson et al. 1999). Another member of this immunoglobulin superfamily, namely CD44, was also upregulated by Fra-2 as well in the scid mouse xenograft tumours and metastasized cells in the lungs as confirmed by Western blot (Fig. 2a) and immunohistochemistry (Fig. 3c). CD44 is a cell-surface glycoprotein with a postulated role in cancer metastasis (Marhaba and Zoller 2004) with binding domains for hyaluronan and other glycosaminoglycans, collagen, laminin, and fibronectin, all components of the ECM (Herrera-Gayol and Jothy 1999).

Overexpression of CD44 has been linked to a number of transcription factors including NFkB and AP-1 (Foster et al. 2000). Most notably, AP-1 has been shown to have a direct effect on CD44 expression by binding the CD44 promoter (Mandal et al. 2011; Smith and Cai 2012). In colon carcinoma cells O-glycosylated CD44 can bind endothelial E-selectin, which in turns contributes to metastasis (Hanley et al. 2005). Following the steps of the metastatic cascade, integrins like ITGB4, which was highly expressed in Fra-2 overexpressing scid tumours, are also O-glycosylated and are suspected to influence the attachment of tumour cells to the ECM as well as cell-to-cell interactions (Oliveira-Ferrer et al. 2017). Moreover, ITGB4 transactivates EGFR/Her2 signaling and promotes lung metastasis in breast cancer cells (Abdel-Ghany et al. 2001; Yoon et al. 2006). It stimulates mammary carcinoma hyper proliferation by inducing phosphorylation and, presumably, activation of c-Jun, the preferred dimer partner of Fra-2 (Eferl and Wagner 2003; Foletta 1996). In agreement with our previous in vitro study (Schroder et al. 2010), we could substantiate the expression of L1-CAM, ICAM-1, and CD44 in the scid mouse model injected with Fra-2 overexpressing cells. Furthermore, we found metastasized cells expressing L1-CAM, ICAM-1, CD44, ITGB4, and TSPAN8 in corresponding lungs of those scid mice (Fig. 3a–e). Mechanistically, the above-named results imply that Fra-2 participates in the metastatic cascade by regulating signaling functions and ligand interactions of these genes.

This special role of Fra-2 also becomes clear in the second experimental approach. We were able to show that the select mice had a survival advantage to the scid mice and a reduced metastatic load in the lung, but nevertheless, tumour growth and metastasis did occur at a later time point. This growth delay can be explained by the lack of E- and P-selectins on the endothelium of the select mice, as the initial contact and adherence of the tumour cells to the endothelium is hampered and thus gives the mice a survival advantage of about 30 days. However, our studies also demonstrate that the transcription factor Fra-2 is able to control the expression of other adhesion molecules as well (L1-CAM, ICAM-1, CD44, ITGB4, and TSPAN8) and thus allows the tumour cells to adhere to the selectin-deficient endothelium via these adhesion molecules. Again, microarray analyses of resected tumours of the select mice were performed to identify possible genes that would assure adhesion to the endothelium in the absence of selectin-binding partners.

**Fig. 5:**
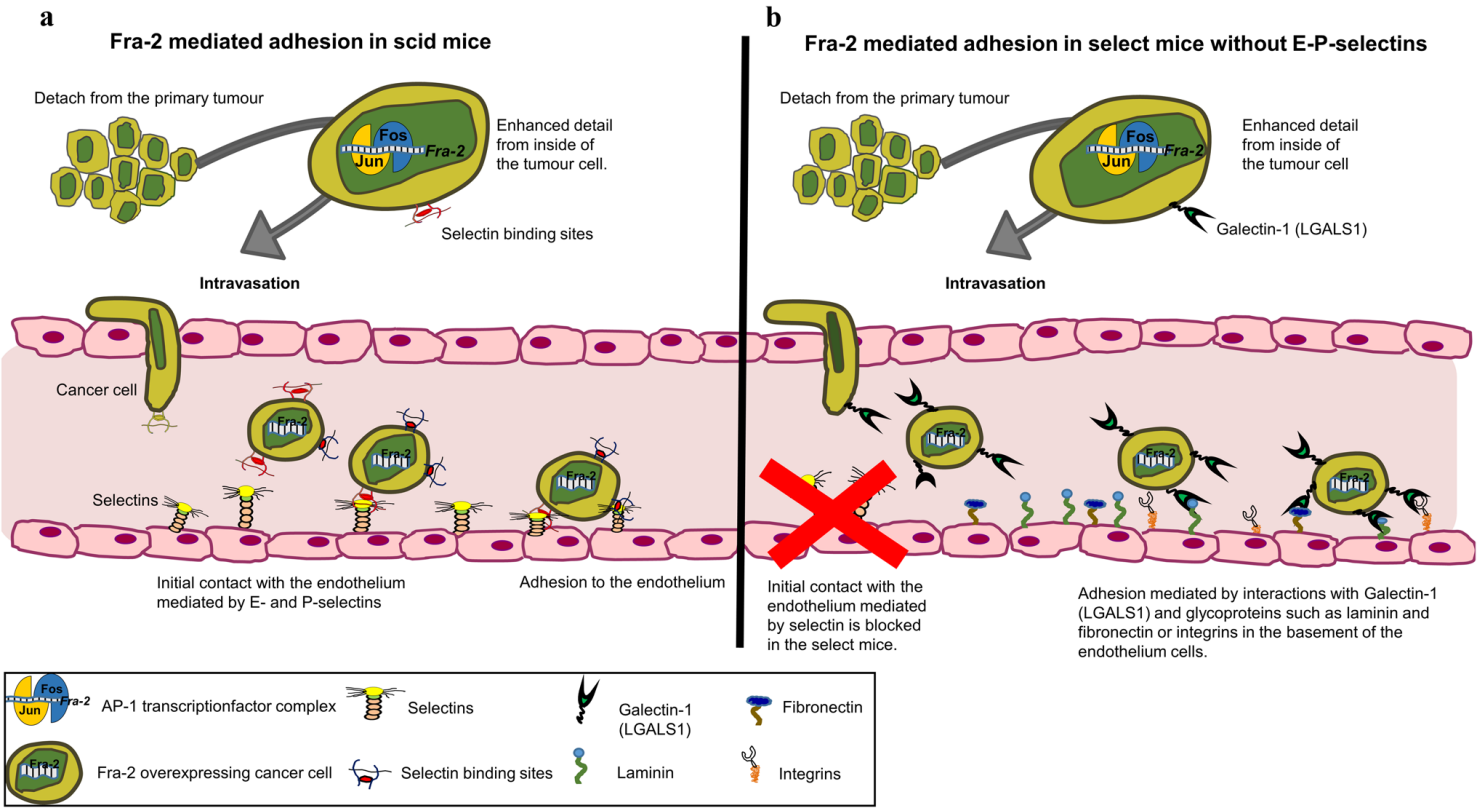
Schematic representation of the main findings of this work: possible mechanism of selectin-dependent in scid mice and selectin-independent in select mice cancer cell adhesion driven by Fra-2. This figure shows the ambivalent function of Fra-2-controlled adhesion in cancer metastasis. **a** When selectins are expressed on the luminal surface of endothelial cells, the Fra-2 induced (over)expression of selectin ligand carrying glycoproteins (L1-CAM, ICAM-1, or also CD44 and ITBG4) leads to an increased number of spontaneous metastases in the lungs and a shorter survival. **b** How this process could take place in a selectin knock-out mouse. Selectins are the first port of call in the leukocyte adhesion cascade, which is however, redundantly organized. Next to selectins integrins and cell-adhesion molecules of the immunoglobulin superfamily follow. This lack of selectins now selects cell adhesion molecules other the selectin ligands on the cancer cells such as Galectin-1 (*LGALS1*), which is also upregulated by Fra-2. The tumour cells presumably come into contact through an interaction of Galectin-1 (*LGALS1*) with fibronectin, laminin, or integrins, and thus mediate the adhesion to the endothelium. This leads to a significantly delayed adhesion and provides the mice a considerable survival advantage

Based on the microarray data, *LGALS1*, also known as Galectin-1, exhibited the most significant upregulation. Its transcript was clearly upregulated in tumours resected from select mice injected with Fra-2 cl 1 as well as cl 2 (Table 4) and we were able to detect the expression of *LGALS1* by immunohistochemistry both in the tumours and in metastatic cells in the lungs of animals of both Fra-2 overexpressing clones (Fig. 4c). Galectin-1 is a non-covalent homodimeric galectin, which preferentially recognizes Galβ1, 4GlcNAC (LacNAc) sequences, which can be present on *N*- or *O*-linked glycans (Elola et al. 2005). Galectin-1 affects the interaction of tumour cells with endothelial cells, which is critical in invasion and metastasis (Kuwabara et al. 2003). Galectin-1 has been observed to arbitrate the adhesion of cancer cells to the ECM; laminin, fibronectin, and other glycoproteins presented in the basement membrane provide the necessary epitopes for Galectin-1-cell-ECM cross-linking (Cousin and Cloninger 2016; Jeschke et al. 2006; Brule et al. 1995). Nam et al. confirmed the binding of Galectin-1 to the cell surface via its interaction with ITGB1 in MDA MB231 cells (Nam et al. 2017). As Galectin-1 expression is at least in part dependent on the constitutive activation of the AP1 transcription complex, it is one more member of cell-adhesion molecules regulated by AP-1 transcription factors (Juszczynski et al. 2007). In summary, this study shows that the transcription factor Fra-2 by virtue of its multiple properties can activate genes involved in cell adhesion at either the receptor or ligand level, thus promoting adhesion to the endothelium and tumour metastasis. This even takes place in the absence of initial molecules, such as selectins, by activation of other downstream genes which launch the adhesion. A possible mechanism of the role of Fra-2 in our mouse model is shown in Fig. 5. Even though Fra-2 appears to be an essential component in the metastasis process through the regulation of several adhesion molecules, it is difficult to envisage Fra-2 as a potential therapeutic target because of its multiple interactions in its function as a transcription factor. Deletion of Fra-2 leads to an early lethality in mice: at birth, knock-out puppies suffer from a severe growth defect and die within the first week (Eferl et al. 2007). Our results furthermore show that the transcription factor Fra-2 plays an ambivalent function in the metastasis of breast cancer. In the presence of selectins, Fra-2 promotes the adhesion of tumour cells to the endothelium due to its influence on the expression of selectin ligands as shown in our in vitro studies (Schroder et al. 2010). Yet, as shown in select mice, Fra-2 can also promote metastasis in the absence of selectins, although more slowly, by activation of selectin-independent adhesion processes. This finding highlights the important role of Fra-2 as a regulator of CAMs of adhesion cascade in breast cancer metastasis.

## Supplementary Information

Below is the link to the electronic supplementary material.

Supplementary file1 (DOCX 3155 kb)

## Data Availability

The datasets generated during and/or during the current study are available from the corresponding author on reasonable request. The raw data were deposited in the Gene Expression Omnibus database (www.ncbi.nlm.nih.gov/geo/) under Accession No. GSE148089.

## Code availability

No code.
