# Supplementary material for: Fra-2 overexpression upregulates pro-metastatic cell-adhesion molecules, promotes pulmonary metastasis, and reduces survival in a spontaneous xenograft model of human breast cancer
Source: J Cancer Res Clin Oncol. 2021 Oct 24;148(6):1525–42. doi: 10.1007/s00432-021-03812-2 (PMC9114065; doi:10.1007/s00432-021-03812-2)
Supplement: Supplementary file 1 — Supplementary file1 (DOCX 3155 kb) [file 432_2021_3812_MOESM1_ESM.docx]

***Supplemental Material***

**Fra-2 overexpression upregulates pro-metastatic cell-adhesion molecules, promotes pulmonary metastasis and reduces survival in a spontaneous xenograft model of human breast cancer**

Sabrina Arnold ^1†^, Jan Kortland ^1†^, Diana V. Maltseva ^2,2*,2**^, Timur R. Samatov ^3^, Susanne Lezius ^4^, Alexander G. Tonevitsky ^2,2*,2**^, Karin Milde-Langosch ^5^, Daniel Wicklein ^1^, Udo Schumacher ^1^ and Christine Stürken ^1, *^

1. Institute of Anatomy and Experimental Morphology, University Cancer Center Hamburg, University Medical Center Hamburg-Eppendorf, Martinistrasse 52, 20246 Hamburg, Germany, (S.A.) arnold.sabrina@gmx.de; (J.K.) jankortland@gmail.com; (D.W.) d.wicklein@uke.de; (U.S.) u.schumacher@uke.de
2. Faculty of Biology and Biotechnology, National Research University Higher School of Economics, Myasnitskaya str. 13/4, 117997 Moscow, (D.V.M.) dmaltseva@gmail.com; (A.G.T.) tonevitsky@mail.ru

^2*^ Shemyakin-Ovchinnikov Institute of Bioorganic Chemistry of the Russian Academy of Sciences, Miklukho-Maklaya str. 16/10, 117997 Moscow, Russia, (D.V.M.) dmaltseva@gmail.com; (A.G.T.) tonevitsky@mail.ru

^2**^ Far Eastern Federal University, Ajax Bay 10, Russky Island, 690091 Vladivostok, Russia, (A.G.T.) tonevitsky@mail.ru

^3^ Evotec International GmbH, Marie-Curie-Str. 7, 37079 Göttingen, Germany

(T.R.S.) timur.samatov@gmail.com

^4^  Department of Medical Biometry and Epidemiology, University Cancer Center Hamburg, University Medical Center Hamburg-Eppendorf, Martinistrasse 52, 20246 Hamburg, Germany, (S.L.) s.lezius@uke.de

^5^ Department of Gynecology, University Medical Center Hamburg-Eppendorf, Martinistrasse 52, 20246 Hamburg, Germany, (K.M.L.) milde-langosch@gmx.de

* Correspondence: c.stuerken@uke.de; Tel.: (0049-40-7410–53574)

^†^ These authors contributed equally to this work.

The file includes: Western blot analyses: original, non-cropped blots, additional information to RNA isolation and cDNA microarray analysis

Figure S1

Full-length blots of image details from Fig 1; human total cell lysates showing Fra-2 overexpression in clones 1 and 2 relative to the MDA MB231 stably transfected cells harboring the empty pIRES-P vector (MDA control). The loading control ß-actin is presented as reblot. The lanes of interest have been framed in black. The others are not related to this experiment.


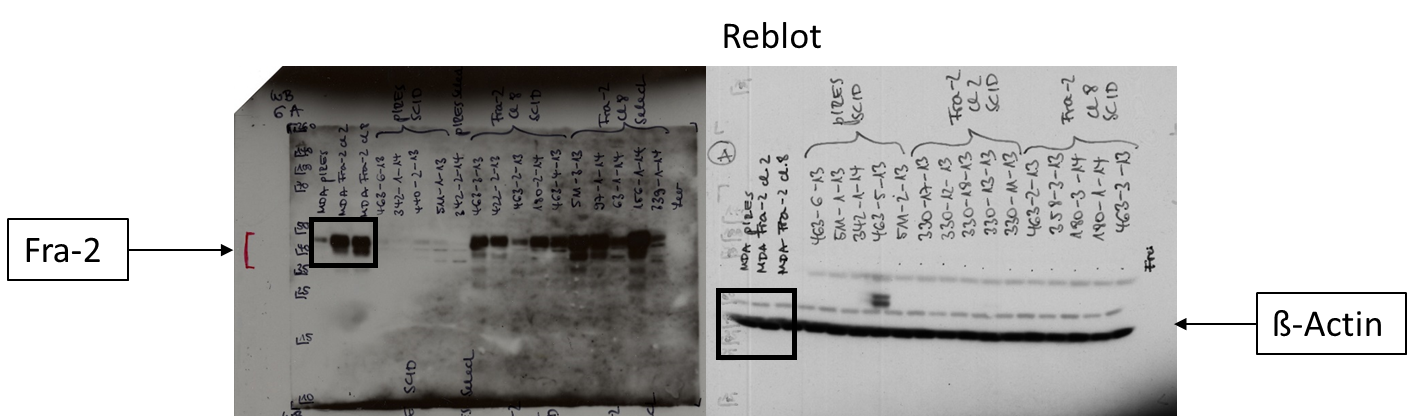


Figure S2

Full-length blots of all used image details from Fig 2; cell lysates of the transfected MDA MB231 cells (first 3 rows) and protein lysates of the resected scid mouse primary tumours of control (second 3 rows), Fra-2 cl 1 (row 8-10, first picture) and cl 2 (row 8-10, second picture) with different staining of Fra-2 (a), L1-CAM (b), ICAM-1 (c), CD44 (d). The loading control ß-actin is presented as reblot. The lanes of interest have been framed in black. The others are not related to this experiment.

a) WB Fra-2


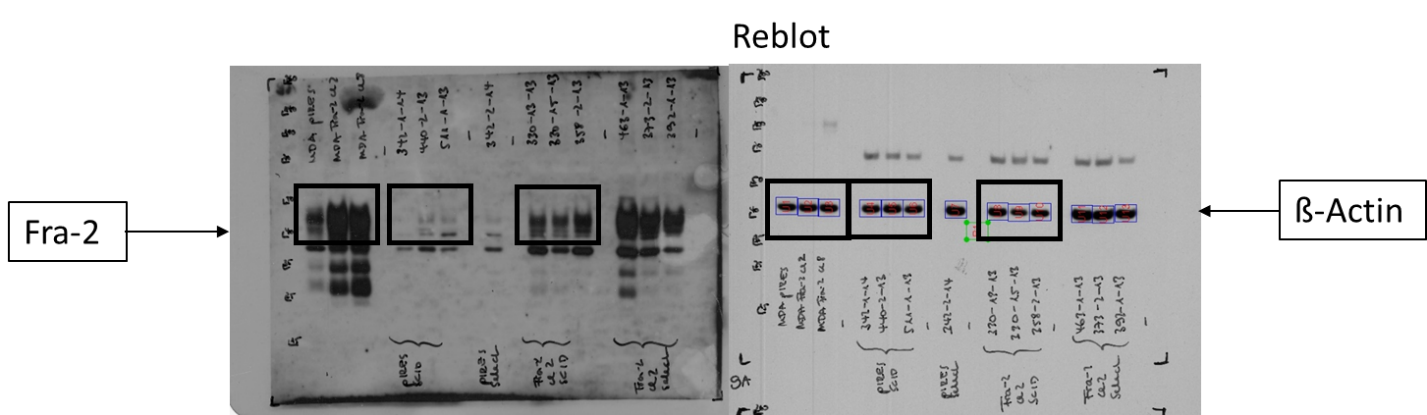


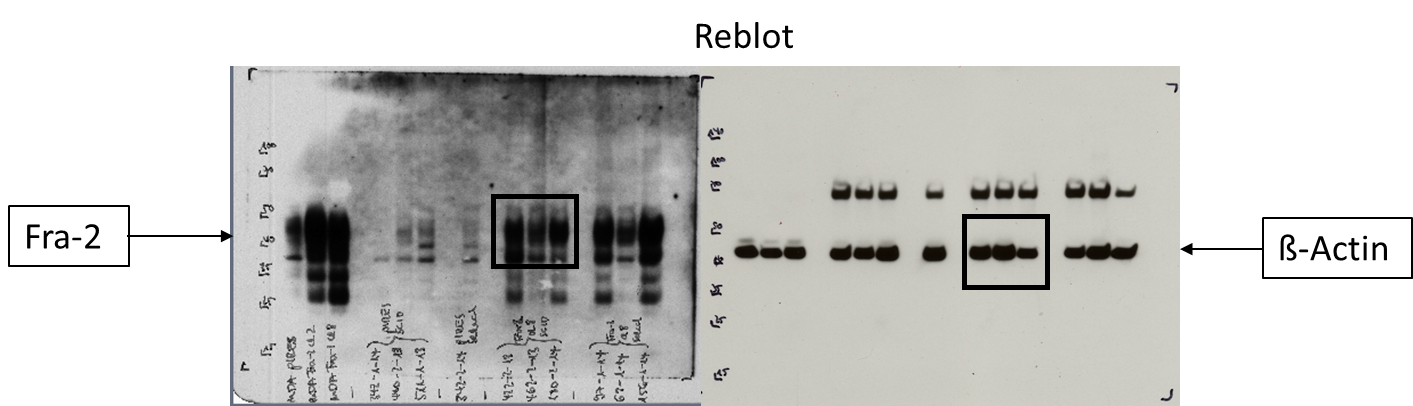


b) WB L1-CAM


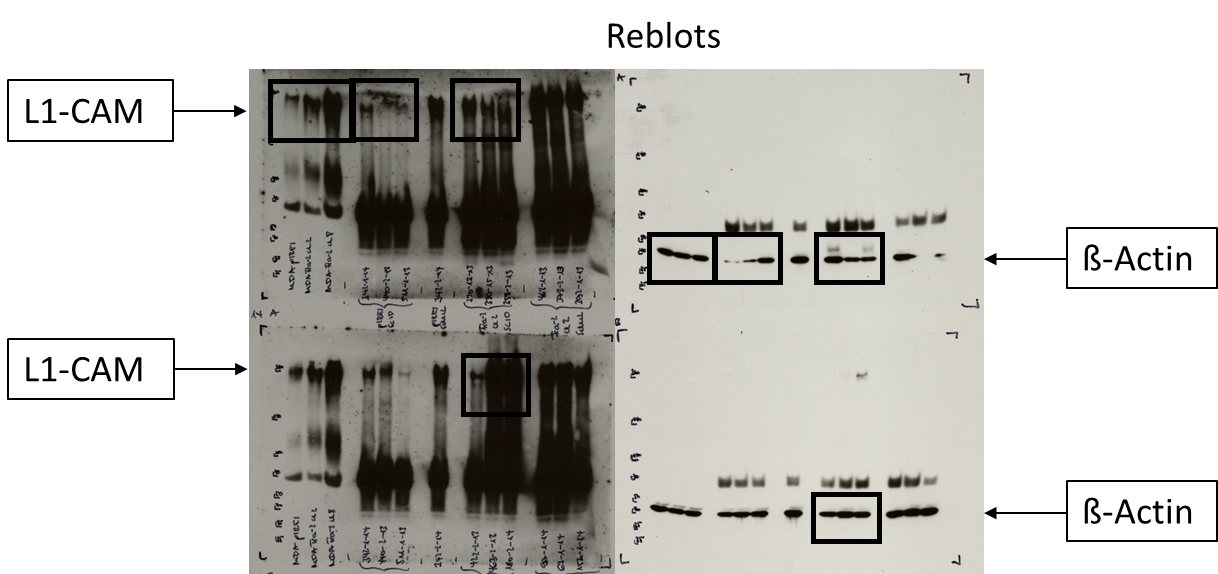


c) WB ICAM-1


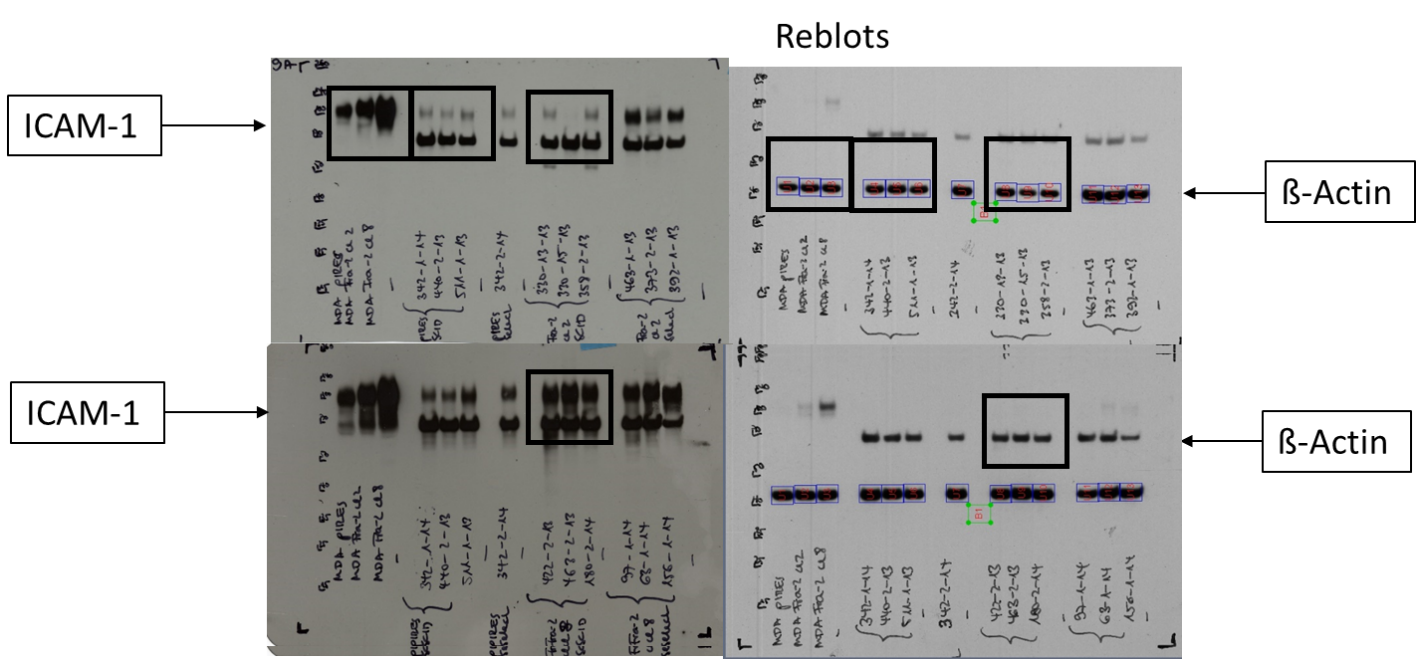


d) WB CD44


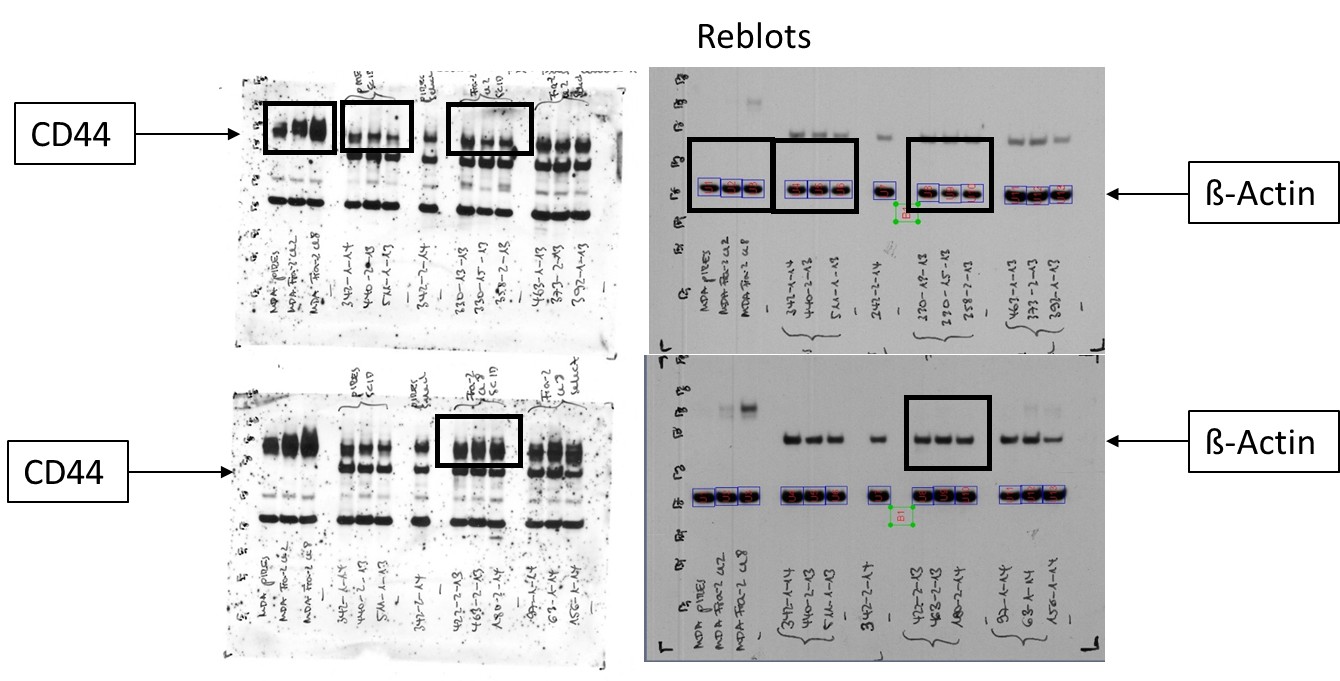


S3

*RNA isolation and cDNA microarray analysis*

Approximately 50 mg of fresh-frozen primary tumour tissue was crushed in liquid nitrogen. The total RNA was isolated using QIAzol Lysis Reagent (Qiagen, Hilden, Germany) and the miRNeasy Mini Kit (Qiagen, Hilden, Germany), according to manufacturer`s instruction. RNA yield was determined by UV absorbance using NanoDrop 1000 Spectrophotometer (Peqlab, Erlangen, Germany). The RNA quality was assessed by analysis of ribosomal RNA band integrity on an Agilent 2100 Bioanalyzer and RNA 6000 LabChip kit (Agilent Technologies, Palo Alto, CA, USA). The RIN values of RNA samples used for microarray analysis were higher than 7.7. The microarray experiments were performed according to the manufacturer's instructions (TermoFisher Scientific UserGuide P/N 703174)^1^. Procedures for cDNA synthesis and labeling were carried out according to the GeneChip WT PLUS Reagent Kit (Applied Biosystems) protocol using 500 ng of total RNA as the starting material. Target DNA fragmentation, labeling, hybridization on Affymetrix Gene Chip Human Transcriptome Array 2.0 microarrays, array washing, staining, and scanning were performed as according to the manufacturer's instructions^2,3^. The raw microarray data (CEL-files) were processed using the Affymetrix Expression Console (build 1.4.1.46) with RMA-sketch method. All CEL-files are available in the Gene Expression Omnibus database (www.ncbi.nlm.nih.gov/geo/) under accession number GSE148089.

S4 Table: Scid functional annotation

S5 Table: Scid select functional annotation

Supplementary References

1 Khaustova, N. A. *et al.* Selectin-independent adhesion during ovarian cancer metastasis. *Biochimie* **142**, 197-206, doi:10.1016/j.biochi.2017.09.009 (2017).

2 Kudriaeva, A. *et al.* The Transcriptome of Type I Murine Astrocytes under Interferon-Gamma Exposure and Remyelination Stimulus. *Molecules* **22**, doi:10.3390/molecules22050808 (2017).

3 Sakharov, D. A. *et al.* Passing the anaerobic threshold is associated with substantial changes in the gene expression profile in white blood cells. *Eur J Appl Physiol* **112**, 963-972, doi:10.1007/s00421-011-2048-3 (2012).
